# Cerebro-Spinal Meningitis

**Published:** 1916-04-29

**Authors:** 


### CEREBRO-SPINAL MENINGITIS.

### SOME OF ITS PROBLEMS.

It is a frequent experience that many questions which propose to the superficial observer a ready and inevitable answer are found to be full of difficulties when tackled by the expert. Many problems in medical science and practice come into this group, and an illustration of them occurs in a disease which has recently attracted public attention—namely, cerebro-spinal meningitis. Most people know that the inflammation of the meninges is associated with the presence of a germ which is known technically as the *Diplococcus intracellularis meningitidis*; they know also that this diplococcus is sometimes found in the mucus of the pharynx. What seem to be natural conclusions follow—namely, first, that the organism must invade the body through the pharynx; secondly, that it must be conveyed thence to the brain. Hence, surely, the solution is to detect the patient when the organism has attacked his throat and to destroy it by antiseptics or other means before it has proceeded to further mischief. The proposal is a plausible one and seems to be within easy and practical reach. But when an attempt to put it into operation is made, difficulties are promptly encountered. Here is an initial difficulty—the presence of the meningococci in the pharynx does not make a patient in the ordinary sense of the term, that is, in this position the germs do not give rise to symptoms, and the invaded person believes himself to be in good health, and to the usual tests may be in good health. He is a "carrier" of the germ, but not a victim of the disease which the germ is capable of causing. Hence when symptoms of cerebro-spinal meningitis appear in the individual the proposal to destroy the meningococci in the pharynx is obviously too late. The steed has already been stolen and the suggestion to lock the door is untimely. From this position there emerges almost inevitably a proposal to examine the throats of all persons who have come into contact with the sufferer. And here what seems to be a very helpful discovery is made, for a certain proportion of these "contacts"—say, roughly, 5 to 10 per cent.—when their throats are properly examined, will be found to yield the meningococcus. Here is surely ground for practical measures. Treat the infected throats and, in future, let the "contacts" with each case of cerebro-spinal meningitis be as few as possible. Easier said than done.

First, on the point just mentioned, is the discovery that when the examination is extended to the throats of a large number of people who have not had any known association with cerebro-spinal meningitis, many "carriers" are again discovered. Indeed, in some of these tests the percentage of "carriers" among "non-contacts" is just about the same as in the "contacts." There follows the obvious conclusion that it is quite as likely that the patient with meningitis has received the infection from the "carrier" as that the "carrier" has acquired the germ from the patient. Hence there is no certainty that isolation of the patient will stop the spread of the disease or, indeed, that it is of any appreciable value to this end. Clearly it is the "carrier" who is mainly to be feared. But from what has just been said the "carrier" is everywhere, or at least he is not limited to those who have had direct contact with patients suffering from cerebro-spinal meningitis. The practical proposal arising out of the preliminary facts stated in this article was that persons having meningococci in their throats should have these destroyed; and the prospect was an alluring one. But it is now seen that so far from treating all these persons it is not possible even to identify them, and, as in a celebrated receipt for making hare soup, it is necessary to catch your "carrier" before you can proceed to cook him.

### Antiseptics and the Carrier.

Still, it may be said that in an institution, or in such an organisation as the Army, "contacts" may be examined, and those who give a positive result may be treated, even though in ordinary civil life such measures are out of the question. Seeing that the numbers will be considerable the task will be a formidable one, but it sounds practicable, more especially as the pharynx is readily accessible for purposes of direct medication as by sprays, paints, gargles, and so on. In this limited measure, at all events, surely something can be done to destroy the meningococci lying harmless, at it were, "in their nests." A few antiseptic applications, and the dangerous "carrier" will be converted into a respectable and harmless citizen. But what is the answer of experience? That success in this direction is most difficult, if not impossible. The "carriers" have been sprayed many times a day with antiseptic solutions, but all in vain. The same solutions applied to the meningococci growing in cultures outside the body are efficacious, but have little or no effect when the meningococci are living in the pharynx. Presumably, therefore, they exist in nooks and crannies which the antiseptics cannot reach. Whatever be the explanation, the meningococci continue to flourish. A second form of attack was the use of vaccines which have for their object the arousing of the natural powers of the body in the hope that these would overcome the meningococci. Once again the result was failure. Indeed, beyond free ventilation and abundant fresh air there is at present no available influence which is known to be capable of causing the disappearance of the meningococci from the throat of the "carrier," and the process usually takes at least two to three weeks. The amount of investigation and experiment necessary to establish even this position has been enormous, and yet it is only a preliminary step to dealing with cerebro-spinal meningitis as an actual disease. The problem sounded simple as a doctrinal statement, but it is seen in practice to be crowded with obstacles.

